# Modern Methods in Bridge Work

**Published:** 1900-05-15

**Authors:** J. L. Young


### MODERN METHODS IN BRIDGE WORK.

BY J. L. YOUNG, D.D.S.

*Mr. President and Gentlemen of the South Western Michigan Dental Society :*—When asked by your Secretary to give you a paper on " Modern Methods in Bridge Work," I hesitated for a while, wondering whether I could write anything of interest on a topic that has received so much attention from those vastly more capable than I, of presenting a subject of so much importance. But, with the hope of calling forth some discussion by presenting small but important points of this work, and thus benefiting all by an intrechange of ideas, I decided to make the attempt. I will, therefore, endeavor to confine myself within the limits of this paper, and not take up your time with the history and development of bridge work.

Notwithstanding my enthusiasm in this work, the day will come, I hope, as the masses become educated to the importance and necessity of dental operations, and the profession becomes so educated and skillful as to successfully combat the ravages of caries, that bridge work will be a thing of the past, for the most skillfully constructed bridge, though the nearest approach to the natural organs of any mechanical appliance, is far from being equal to them.

The successful bridge worker must be an expert mechanic and use good judgment in selecting cases. He must discard the use of bands or open faced caps for attachments to abutments or piers, for the finished piece can not possibly be stronger than its weakest part. The architect would soon come to grief if he were foolish enough to rely on soft clay or shifting sand for a foundation on which to place an abutment or pier to construct a bridge, and so in this branch of our chosen profession the abutments and piers must be capable of carrying the strain of the finished piece. And the attachments to them must be so constructed as to overcome all liability of solution of cement which unites the two as one solid piece. Then the spans or dummy teeth must also contain sufficient material to withstand the strain of mastication.

It might be well to consider here the possible strength of the muscles of mastication. According to Black, it ranges from thirty to one hundred and seventy-five pounds in the incisor region and from sixty to two hundred and seventy in the molar region. While this may seem excessive force, it is the opinion of the writer that it is far below the possible under favorable conditions. This being the case, what must happen to a narrow band of 30 gauge, 22 k. gold, (which can easily be bent out of shape between the fingers) when used as an attachment to a cuspid supporting a bridge extending to the first or second molar, saying nothing of the liability of solution of cement and inception of decay beneath the band around the tooth? Furthermore, a band must accurately fit the neck of the tooth at the upper edge of the enamel beneath the free margin of the gum, or irritation and recession of the gum will follow. In order to admit of this, the enamel in most cases has to be entirely removed from both proximal sides, which leaves an unsightly appearing tooth no matter how well covered with metal, and, if the exposed dentin is not protected, decay is almost a certainty. Attempts at anchorage by filling around dove-tailed bars must prove a failure, for, in teeth with vital pulps, the length of bar must necessarily be very short, while in pulpless teeth, though the bar may be of sufficient length, it is a well known fact that they are very liable to break away around the filling, and, should caries recur in either case around these anchorages, the presence of the bridge renders the proper filling of such cavities impossible. It must likewise be apparent to all of us that where the teeth are short, say 1/8 inch between ridge and opposing teeth, any attempt at full porcelain restoration must end in failure.

At this point it might be well to cite a special case of porcelain restoration for a patient who had worn a plate for twenty years carrying the upper incisors. The tissues had absorbed to such an extent that it required 1/4 inch of porcelain above the necks of the incisors to restore the contour of the face. The cuspids which were used for abutments were very short, and thus necessitated a combination of metal and porcelain. This was accomplished by using the Mason detachable facings, the backings being constructed of iridio-platinum which were soldered together with pure gold. To these backings was soldered a piece of 30 gauge platinum, previously swedged to fit on space between cuspids to be restored with porcelain. The body was then fused on, the facings ground to fit and cemented in place with chloro-percha and the finished piece cemented firmly in place, restoring the lost teeth and reproducing the original expression, the pink porcelain so closely imitating the adjoining gum as to be scarcely noticeable.

In all lower bridges back of the cuspids all-gold dummies are to be preferred, as they do not show and are more substantial, and where the bite is very close it is good practice to supply only the occluding surface, making them sufficiently thick to withstand the strain of mastication, leaving a good free opening between them and the gum. In this way we certainly can supply the lost organs in such a way as to be more easily kept clean than the natural ones, and after a short time the space causes little annoyance to the patient.

Properly speaking, a bridge must have two abutments, but this does not always apply in dental bridge work.

For example, a missing lateral can be successfully sup ported by the cuspid, or a second bicuspid facing by a first molar. Most all other cases must have two or more attachments.

As we have a great many systems of bridge work, it is the opinion of the writer that it is best to select one of these and become so accustomed to its use as to be able to apply it to any case. The Morrison system, with some modifications, has been used by the writer for some years with good results.

The attachments to be relied upon are the full gold cap and the so-called Richmond crown, the latter to be used where too much show of gold would be unsightly. To properly cap a tooth or root it is essential that it be so shaped that the circumference at the cementum margin be at least as great as at any point between it and the occlusal portion of the tooth. Where it is possible, it is even better to so shape the tooth that the circumference just beneath the free margin of the gum is considerably in excess of any other point. To show how to do this on paper is very simple, but to perform the operation in many cases is very difficult and often is the cause of failure in an otherwise good piece of work.

It might not be amiss to give here the method of the writer in constructing a bridge running from the cuspid to the second molar superior, using a full cap and porcelain-faced crown for attachments.

If the outlines of the molar are complete or can be restored by filling, take an impression in modeling compound in two parts (using this little tray made of a brass ferrule ¾ inch in diameter, sawed in halves, to which is soldered a small brass hinge) using soapstone to cause the compound to separate. A measure of the neck of the tooth is then obtained with binding wire and cut open in order to remove it. This is used to gauge the size to trim the cast to be run in plaster in this impression. When the plaster is thoroughly set, remove the compound and trim cast so as to get up above neck of tooth, using measure taken as a guide, and, as the outside of the gold cap will be the same size as the cast, we must allow for the thickness of gold at this point as it is not necessary to reduce the size of the neck of the tooth. In the box and screw appliance of the Morrison system place sufficient mouldine to half fill it. In this is half buried either proximal side of cast of tooth, the square end looking rootwise, being against one side of box. Melotte's metal is now melted and poured in, and as soon as crystallized the mouldine is removed and the surface of the metal, while still warm, coated with yellow ochre paint. While this is drying, more of the same metal is melted and then allowed to cool till it commences to stick to the side of the spoon, when it is quickly poured in—giving the other half of the impression. When this is cold it can be readily separated and the cast removed. In this metallic impression can be swedged a seamless gold cap from a blank previously drawn up with the Morrison system of 22 k. 28 gauge gold, exactly reproducing the natural tooth. The cusps are filled in with 22 k. solder to strengthen it, and it is now ready to adjust as soon as the tooth is trimmed down at the next sitting. A full cuspid cap can be made in the same way when desired.

The next step is the very important and often very painful one of removing the enamel from the tooth, which, in the majority of cases, is absolutely essential if the cap is to be properly fitted. Cocain can be used to good advantage by means of the cataphoric appliance, and, in many cases, after the dentin has been exposed in several places on the occlusal surface and the cocain applied in this way, the remaining enamel can be removed without causing any pain. A true, knife-edged carborundum disk in the engine is very effective for this work, for we must remember the fewer dental tubuli we strike at once the less pain we cause, so when a disk wears thick it should be put in the lathe and trimmed thin and true with a diamond tool. The occluding surface should be removed until we have at least 1-32 inch between it and the nearest point of the occluding teeth. With a properly shaped diamond disk kept wet, trim the proximal surfaces, slightly slanting toward the occlusal surface. With a small, true carborundum, slant the buccal and lingual walls as the proximal ones. Now comes the hard part, especially if the third molar is in position, to properly shape the disto-buccal and disto-lingual corners, and also to remove the bulge of enamel at the gum margin on the lingual and buccal surfaces. A diamond, disk-swedged saucer-shape, is of great service in this work, as it is a rapid cutter when kept wet and does not wear thick nor injure the gum. Pointed cross-cut burs, made by Ivory, for both direct and right angle hand pieces are indispensable. After once using these rapid cutters, the Case enamel cleavers and chisels will be entirely discarded for this work. With binding wire take a measure well above free margin of the gum, and, if tooth has been properly trimmed, the wire loop can be easily removed without the aid of any instrument, as the circumference at the neck is greater than at any point between it and the occlusal aspect. This important test should never be omitted.

The cap is then pressed on the tooth with the fingers and the approach to gum margin and occlusion noted, the top of cap trimmed to conform to gum line and to such an extent as to admit of proper occlusion, and then beveled from without in, so as to readily pass between the free margin of the gum and neck of tooth, usually about 1-32 inch. When these precautions have been taken and the cap forced on the tooth, the result is a perfect fitting, accurate, occluding and knuckling reproduction in gold, causing no irritation of the gum or other inconvenience. It is well to remember that if the patient complains of the cap being too tight, it is too loose and causing pressure on the soft tissues, for it is absolutely impossible to force a cap on so tight as to cause pain by its pressure on the tooth.

If the cuspid possesses a vital pulp and it is decided to use a Richmond crown, with a diamond disk nick the tooth on both lingual and labial sides on a line with the gum margin on the mesial side, and then adjust rubber dam and with excising forceps remove the crown, exposing the pulp. Having previously prepared the hypodermic syringe with a solution of cocain, immediately insert the needle into the pulp, and, at the same time, force some of the solution into it. In this way the needle can be forced well up and the pulp thoroughly numbed with very little pain, as it has not yet recovered from the shock of excising the tooth. With a fine Donaldson cleanser remove the pulp and if hemorrhage can be controlled, and it usually can with 25 percent pyrozone, the root may be filled at once, by first using campho-phenique and then chlora-percha and gutta-percha cones, entirely filling the canal. In reaming out the canal for the post, the gutta-percha acts as a guide and lessens the danger of perforating the root.

On removing the dam, the root can be trimmed down by the use of saucer-shaped diamond disks and the different root trimmers so as to be slightly cone-shaped, the measure of root taken, wire removed and cut open so as to give the length to cut band of platinum 28 gauge. The width of this depends upon the root, but ⅛ inch is usually sufficient. The ends should be beveled from opposite sides, so when brought together they lap, and then soldered together with pure gold. This band is then fitted to the root, using the same precaution as with the molar, to have it conform to the neck and pass beneath the free margin of the gum. Remove band, and with true carborundum wheel (the only place a wheel should be used in this work) bevel end of root until labial portion is level with or slightly beneath the free margin of the gum, which can be easily done as the rubber dam has pressed it back. Re-adjust band and trim to come flush with end of root, to this is soldered the cap of pure platinum No. 30 gauge. The cap is placed on root and a hole punched with sharp-pointed instrument into the pulp canal to receive the post which should be of iridio-platinum No. 13 gauge. The root is now reamed out, the cap re-adjusted and the post, which has been slightly tapered, forced through the hole in the cap, and as this turns edge of cap around the post into the pulp canal it gives bearing enough to hold the post accurately in place while soldering. Mark the post just below cap with a sharp excavator on labial aspect, remove post, then the cap and place together in relative positions and solder with pure gold; and at the same time flow a little pure gold over the labial aspect of band, so in case it does show any it will present that beautiful golden color our patients all like.

Cut the post off to admit of proper occlusion and on both abutments adjust attachments, which, if properly constructed, should set real firm and yet be readily removed in the plaster impression to be taken. Take bite in modeling compound, using bite tray made by the Detroit Dental Mfg. Co., and impression in plaster. Both of these should include the adjoining incisors. The cuspid root should now be covered with gutta-percha base plate to prevent gum from falling over that part cut off beneath the gum margin and thus rendering the adjustment of bridge painful. This is easily done by using a small tack covered with sufficient gutta-percha that when warmed it will fill the pulp canal and spread out over end of root under pressure of the finger. The rubber dam should now be adjusted on the molar and this coated with a saturated solution of silver nitrate until dentin turns quite black, thus closing up the dental tubuli, rendering the tooth much less sensitive to thermal changes and the action of phosphoric acid of the cement. The dam is then removed and the patient instructed to rinse the mouth with a saturated solution of chloride of sodium to prevent the disagreeable metallic taste following the use of silver nitrate.

The cap of the cuspid attachment is now filled with Mason's anti-flux to permit it to be readily removed and re-adjusted to the cast. The cast is now run of material sold as investment plaster by the Detroit Dental Mfg. Co., as it sets very hard and strong and does not shrink with the heat required in soldering the piece. As it sets somewhat slow, it is well to leave it stand over night before separating. When separated it is mounted on articulator (Gritman) and the bite filled in with plaster in the usual way, and when set the modeling compound is removed. The cuspid attachment is now removed from cast, which is easily done by cutting in so as to insert point of knife above end of post. In this way the cap can be removed and re adjusted as desired in finishing the work, doing away with the necessity of a second impression, etc.

The post is now cut flush with the exposed surface of cap, and labial edge of cap beveled as much as the thickness of the metal will allow, and replaced on cast. A properly selected plate tooth is ground to fit, having it at least 1-32 inch shorter than the finished crown is to be. This facing should be beveled from very near the pins to the upper labial edge, and should have a short bevel at the point so as to allow the pure gold backing, 32 gauge, to cover all but the labial and proximal aspects. Over this is fitted a backing of iridio-platinum, 28 gauge, allowing it to extend root wise just enough to admit of holes to accommodate the pins, and it should extend 1-32 inch beyond the incisal end of facing. The pins are now nicked with a sharp knife to hold the backings in place, and wax placed in the V-shaped space between the gold and platinum backings at the incisal edge. The facing is coated with yellow ochre paint to prevent borax from getting on it and causing it to check. Another cause of checking is the contraction of solder in cooling and is more liable to occur when low carat solder is used. The tooth is now invested, wax removed and 22 k. solder flown in, uniting the two backings and giving a very strong occluding edge, which is more essential in the upper cuspid than with any of the other anterior teeth. Why not remove the facing for this soldering?—Ed. Incisor dummies or crowns can be protected in the same way. The tipped facing is now trimmed up and waxed in place on the cap, the whole removed from cast and invested, using paint as before, the wax washed out with boiling water and the two united with 20 k. solder by heating the investment from underneath till the first piece of solder flows and thus form a perfect union with the gold backing on facing and the cap, entirely doing away with cracking of facing which is caused by the expansion of the pins if the porcelain is not heated first. When cold it is pickled in muriatic acid, which dissolves the borax, polished and replaced on cast, and, if necessary, ground to articulate.

Suitable porcelain facings are now selected for the dummy teeth, being careful not to have them too long and especially not too wide, as it is better to have a little space between the facings than to have to grind them or have them check in the final soldering. As the same principles are involved in the construction of these, it will be necessary to describe only one, say the first bicuspid. Sufficient 22 k. scrap is melted up, and while hot placed in an S. S. White cusp plate, a large steel punch placed on it and the gold driven with a heavy hammer. In case it should stick in the plate, it can be easily removed by light taps of an ordinary hammer on the opposite side of the plate directly over the gold. This being of solid gold (no solder), it can be cut and trimmed to give the exact occlusion required with no danger of change of shape in soldering. The labial side of the solid gold cap is cut on a 45° miter, and the incisal end of the facing ground to fit it. The facing is now backed with pure gold, 32 gauge, extending from the bevel above the pins to the labial edge. Over this is placed a backing of platinum, 30 gauge, from bevel to bevel, and pins nicked as before. The facing and gold cusps are attached with wax, invested and soldered with 22 k. solder, using just enough to unite the two. When cool, remove from investment and place on articulator, along with the other dummies, using just enough sticky wax to unite them all together. An iridio-platinum bar is now cut from 13 gauge round wire, and passed through rolls to make it 15 gauge. This should be long enough to reach from molar cap to the cuspid crown and placed in position, and sticky wax flowed around it to unite wire, dummies and attachments altogether. Any wax extending onto porcelain should be removed, and the case invested for the final soldering, the porcelains being covered with paint as before. The wax is removed, and at least 18 k. solder flowed in uniting the bar, dummies and crowns in one solid piece. The object of this bar is to give strength, to aid the solder to flow from one dummy to another, and to prevent shortening of the piece by contraction. When cool, grind and polish, being careful to leave no pockets for lodgment of food.

The bridge is now tried on to see that all is right, and, if found so, is ready to be cemented in place. In doing this, the abutment teeth should be kept as dry as possible by using absorbent cotton rolls or napkins, the teeth washed off with alcohol and the gum coated with equal parts of aconite, iodine and chloroform, which lessens the irritation of the phosphoric acid of cement. The cement, Justi preferred, is mixed to the consistency of thick cream, a little placed in the cuspid root with a properly shaped piece of orange wood, and sufficient placed in the caps to have some escape, as the bridge is forced on with pressure and afterwards tapped home with mallet and driver. When the cement has set, the surplus should be removed from beneath the free margin of the gum and syringed away with a strong solution of sanitol and warm water to further relieve the irritation. It is well to have the patient return in a few days to see that there has been no cement left beneath the gum margin, and also to see that the occlusion is right, as many otherwise good bridges are ruined by mal occlusion.

The following points are important in the work:

1. Investments should be as thin as possible so long as strong enough to hold parts in relative positions, and should not shrink.

2. Fine coal ashes and plaster, equal parts by weight, make a good investment, and if necessary a little asbestos fibre may be added.

3. The piece to be soldered should be heated from the porcelain aspect till the first piece of solder flows.

4. Where porcelain is used the soldered piece should be cooled gradually, which is very successfully done by wrapping the hot investment in paper and placing it in a closed vessel.

5. The borax slate should be clean, and the borax rubbed into a cream with water, and just enough used to cause the solder to flow where wanted.

6. Borax can be removed from porcelain and gold by boiling the piece in commercial muriatic acid, full strength.

7. The facings can be protected from borax by coating them previous to investing with a paint composed of yellow ochre, 4 parts, boracic acid 1 part, mixed with boiling water.

8. Diamond disks wear well when kept wet, and can be swedged to desired shapes.

9. To keep disks and stones wet and also to hold tongue and cheek out of way, use old mouth mirror, from which the glass has been removed and a wire soldered across to hold a small sponge to carry the water.

10. The more perfect the articulation of a bridge the less danger of trouble with the abutment teeth.

The writer has frequently been taken to task for cutting down perfectly sound teeth in order to fit attachments for a bridge, particularly where only one tooth is lost, as first lower molar. Is it not better to supply the lost organ, and, in so doing, forever protect the adjoining teeth from decay, than to allow the teeth to separate or tip over and interfere with proper occlusion, probably causing exostosis? Or to insert a plate to cause irritation of the gum and abrade the adjoining teeth, saying nothing of the annoyance and discomfort? If bridge work is good at all, it is in just such cases. In the opinion of the writer, a tooth properly capped under ordinary conditions is not liable to be troubled with decay if the pulp be alive, and if the pulp be dead there is no better way of preserving the tooth.

The extent to which bridge work may be employed depends very largely on the conditions of the supports. Where teeth are firmly set and the surrounding tissues healthy, the cuspids and second molars will successfully support a bridge of fourteen teeth. Where a bridge has but two attachments, it is well to have the intervening teeth as near the straight line as possible, and thus do away with lateral strain. The third molar can often be used to good advantage as an abutment, and, if firmly set, will successfully carry a span to the cuspid of the same side.

DISCUSSION.

Dr. White: I can heartily agree with the essayist that failures in bridge work are too often the result of neglect to secure proper attachments and the use of sufficient material to make them withstand the strain put upon the bridge in use. The trouble comes from the fact that we are trying to make bridges at the prices of rubber plates. It is not only important that we use a good method, but the work must be honestly made. Much time and skill is required to construct a practical bridge, especially to replace more than one or two teeth. I am not an enthusiastic bridge man. I do not believe in constructing a full bridge and attaching it to three or four roots, as is too often done, especially when these roots are not firmly fixed in the process. In the front of the mouth a bridge to replace as many as four teeth is often a success. But in the molar region even a bridge of two or three teeth needs good, solid roots for supports. In fact, removable bridges or plates are, in my judgment, preferable in these locations. I trust the time may come when we shall not be compelled to make either plates or bridges.

Dr. Rowley: I should like to ask Dr. Young if he thinks it advisable to cut off solid crowns in all cases when using the cuspids as supports?

Dr. Young: I will in no case rely upon an open-faced crown or a band as an anchorage for a bridge. For a man having a mustache to hide the crown I would make an all gold shell crown for the cuspid attachment, otherwise I would cut off the crown and make a porcelain faced crown for the attachment.

DR. RIX: I have made considerable bridge work, and my experience has been similar to that of Dr. Young. I would never attach a bridge of any considerable number of teeth to an open band. I have seen such bands that had slipped up on the teeth and the gums were in a very bad state as a result of the impingement of the bridge. It requires very good judgment indeed to know what kind of attachment to make in some cases. I prefer to cut off the crowns even though without carious cavities. This can be easily and quickly done by the usual methods, and the pulp removed at once by driving a hickory peg, which has previously been dipped in carbolic acid, into the pulp canal with a quick, sharp blow. Of course, this method is only applicable to the anterior single rooted teeth. This gives a good root which can be utilized as the demands of the case suggests and makes a difficult operation at once easy, and in the end secures a good result.

DR. ESSIG: Dr. Richardson, of Chicago, makes what he calls a slipper crown, which is an open face or sometimes called window crown, which serves a good purpose and does not necessitate so much mutilation of the sound teeth.

DR. SIDDELL: I have experienced some trouble in taking impressions and bites for bridge work. This, in some cases, I have overcome by taking the bite tray made by the Detroit Dental Manufacturing Company, and sawing off the inner guard, and, when ready to take the impression, re-attaching it with strong, adhesive wax. Then I take the impression with plaster, and when the plaster has set in the mouth I break away the lingual section and remove it from the mouth, this relieves the buccal section so that it can be easily removed. The parts are then re-assembled and waxed together and the impression is ready to be poured.

Another item that has served me a good purpose in preventing injury to the plaster models by the frequent and necessary re-adjustment of crowns for bridges, is to make little copper ferules from very thin sheet copper, say 32 gauge, and after the caps which are to be used for abutments are made and placed in the mouth and the impression taken to secure a proper position on the cast, so that the dummy teeth may be properly ground and adjusted, by placing these ferules inside of the abutment caps, adapting them to a snug fit before pouring the impression. Then when the impression is poured, the abutment caps will be easily removed from the plaster teeth which are bound or covered by the copper ferules, so that the bridge abutment caps may be replaced or removed as often as necessary without marring the tooth, and the copper-bound tooth will always compel the placing of the cap in exactly its right relation and position.

I use plaster and whiting in equal parts, and find this investment will not shrink or crack and will withstand the heat admirably.

I have the patient take a mouthful of cold water and hold it in the mouth to prevent the after pains from setting a bridge.

I use cocain by the pressure method to anesthetize pulps, and follow it with carbolic acid to control the hemorrhage.

Dr. White: I have used the chloride of ethyl a great deal as a local anesthetic in bridge work, and very satisfactorily.

I don't, as a rule, set permanently a bridge until the patient has worn it, set temporarily for a day or two, so that the articulation may be made more naturally or that any changes which may be necessary can be made out of the mouth.

Dr. Hoff: Every one seems to "taboo" the open face crown. I think there are cases where it would be entirely wrong to sacrifice a good tooth for the purpose of setting a bridge, I don't believe it is possible for any one to reproduce the natural tooth in all its artistic, to say nothing of its useful functions, by ever so cleverly made artificial crown, and the tooth itself will assuredly have its life shortened by such treatment. I have made open face crowns for cuspids and bicuspids for bridge attachment that are infinitely more artistic (and equally useful) than any porcelain faced crown that could be made. It is possible that an all porcelain crown would not be so objectionable esthetically, but it would not serve as a bridge attachment. I think the trouble in this matter comes from the fact that no judgment is exercised in selecting patients for such operations. I would never put an open faced crown on a tooth for a patient who would not keep his teeth clean; but in the mouth of a person who will take the care to brush his teeth there can be no objection to a properly adopted open faced crown. Again, an open faced crown is not as strong as a full cap or porcelain faced crown, and where strength was necessary I would use the stronger crown. We should cultivate the habit of making a complete diagnosis of every case, and vary our methods to meet the requirements of individual cases. Definite rules can not be made which will govern the construction of bridge work any more than for filling teeth or treating pyorrhoea alveolaris.

Dr. Young: In closing, I would say, that we need to be, above all things, honest, and do this work as well as we know how. The temptation to slight it for a low fee, or for any other reason, should never influence our judgment.

A very important matter to take into consideration is the occlusion of the teeth. Be sure that the bridge is adjusted in this respect. To obtain this every bridge, however small, should be made on models in either a Gritman or Bonwell articulator.

I would not hesitate to make a fourteen tooth bridge if the occasion warranted it, and I had at least four good anchorages.

My failures have all come from poor construction. I did not use sufficient material and in the proper way. I think where one open crown will be a success ninety-nine will fail.

